# Malnutrition and Its Determinants among Older Adults Living in French Caribbean Nursing Homes: A Cross-Sectional Study

**DOI:** 10.3390/nu16142208

**Published:** 2024-07-10

**Authors:** Maturin Tabue Teguo, Laurys Letchimy, Leila Rinaldo, Michel Bonnet, Huidi Tchero, Nadine Simo-Tabue, Denis Boucaud-Maitre

**Affiliations:** 1 Equipe EPICLIV, Université des Antilles, 97233 Fort-de-France, France; nadine_tabue@yahoo.fr (N.S.-T.); denis.boucaud@gmail.com (D.B.-M.); 2 Centre Hospitalo-Universitaire de Martinique, 97261 Fort-de-France, France; laurys.letchimy@chu-martinique.fr (L.L.); michel.bonnet@chu-martinique.fr (M.B.); 3 Inserm U1219 Bordeaux Population Health Center, University of Bordeaux, 33405 Talence, France; 4 Centre Hospitalo-Universitaire de Guadeloupe, 97110 Pointe-à-Pitre, France; leila.rinaldo@chu-guadeloupe.fr; 5 Centre Hospitalier Louis Constant Fleming, 97150 Saint Martin, France; h.tchero@chsaintmartin.fr; 6 Centre Hospitalier Le Vinatier, 69500 Bron, France

**Keywords:** nursing homes, older adults, malnutrition, Caribbean

## Abstract

Background: This study aimed to assess the prevalence of malnutrition and its determinants in older adults living in French Caribbean nursing homes. Methods: This cross-sectional study was taken from the KASEHAD (Karukera Study of Ageing in EHPAD) study. Nutritional status was assessed with the Mini Nutritional Assessment Short-Form (MNA-SF). Clinical characteristics and scores on geriatric scales (Mini-Mental State Examination (MMSE), Activities of Daily Living (ADL), Short Physical Performance Battery (SPPB), Center for Epidemiologic Studies-Depression (CESD) and Questionnaire Quality of Life Alzheimer’s Disease (QoL-AD)) were extracted. Bivariate analysis and logistic models adjusted were performed to test the association between nutritional status and both socio-demographic variables and geriatric scales. Results: A total of 332 older adults from six nursing homes were included in the KASEHPAD study. Among the participants, 319 had an MNA-SF score. The mean age was 81.3 ± 10.6 years, and half of the participants were men. The frequency of malnutrition (MNA-SF ≤ 7) was 27.6% (95% confidence interval (CI): 22.0–32.5) (*n* = 88). Based on the multivariable analysis, a low MMSE was associated with malnutrition (OR: 0.81 (0.68–0.92); *p* = 0.015) and there was a borderline significant link between a higher CESD score and malnutrition (OR: 1.05 (1.00–1.12); *p* = 0.07). Conclusions: Cognitive decline and a tendency toward depression were associated with malnutrition in nursing homes in the French West Indies. Although this study cannot establish causal relationships, the identification of these three geriatric syndromes in nursing homes is crucial for preventing adverse health events.

## 1. Introduction

Malnutrition is a pathological condition characterized by a persistent mismatch between the metabolic needs of the body and the availability of energy [1]. In older individuals, malnutrition is considered one of the main geriatric syndromes, representing a clinical condition rather than a specific illness [2]. It is defined as a state resulting from alterations in nutritional intake or energy expenditure, leading to a decrease in lean mass and/or body cell mass [3,4,5]. As with many geriatric syndromes, malnutrition is multifactorial in origin (social, psychological, environmental and/or financial vulnerability). It can both result from or contribute to various diseases (e.g., stroke, diabetes, heart failure, infectious disease, cancer, metabolic disorders, renal insufficiency, pulmonary disease, digestive or thymic disorders) or clinical conditions such as thinness, major neurocognitive disorders or sarcopenia [6,7,8]. Commonly, older adults with malnutrition do not seek medical assistance for this reason. In this context, malnutrition is considered a situation that should be addressed by secondary prevention programs [2]. Screening for malnutrition and its subsequent management should be undertaken proactively, as malnutrition is an important prognostic marker in older adults. It predicts adverse outcomes such as falls, impaired quality of life, unscheduled hospitalisations, delayed wound healing and mortality [9,10,11]. The prevalence of malnutrition increases with age and varies according to the place of residence [12]. In France, the prevalence of malnutrition is estimated at 6.4% and 18.5% in rural and urban areas, respectively [13]. Depending on the diagnostic criteria and tools used [14,15], the prevalence ranges from 4 to 10% among persons living at home and from 15 to 38% in residents of long-term care facilities (LTCFs), as assessed by the Mini Nutritional Assessment Short Form (MNA-SF). This screening instrument identifies individuals at risk of or suffering from malnutrition among community-dwellers and LTCF residents [16]. Undernutrition represents a significant concern for the majority of nursing homes, with a number of establishments having established meal planning committees. The committees convene with the objective of devising menus. Moreover, the majority of nursing homes employ a dietician. The establishment of menu committees in nursing homes provides residents and their families with the opportunity to participate in menu planning in collaboration with the establishment’s staff. Such meetings provide a forum for residents to express their preferences and suggestions for improvement. The development of menus is typically aligned with a six-week food plan. Even in a mass catering setting, efforts are made to accommodate local preferences, such as offering soup at each meal and cold meats once a day. Furthermore, in the event that the establishment in question is engaged in a collaborative endeavour with an external service provider, the latter may be extended an invitation to participate in the aforementioned committees.

In the healthy aging programme proposed by the World Health Organization for the period 2019–2030, primary prevention via the ICOPE program identifies the six intrinsic capacities necessary for healthy aging [17], with nutritional status being one of these capacities. 

The French West Indies are undergoing accelerated population aging, with an anticipated decline in the overall population. Currently, inhabitants aged 60 years and over represent nearly 30% of the total population [18]. This population presents certain specificities compared to mainland France, notably a higher prevalence of dementia, specific Parkinsonian syndromes probably linked to environmental factors, metabolic syndromes, social isolation, financial precarity and higher dependency rates [19,20]. One consequence of the accelerated aging of the population is the rising number of dependent older adults living in LTCFs (with around 3000 LTCF places in the French West Indies).

Long-term care facility (LTCF) residents exhibit a number of clinical and geriatric characteristics that distinguish them from the general population. For instance, when compared with individuals who live in the community, there is a higher prevalence of dependency, cardiovascular pathologies (such as hypertension, diabetes and stroke) and geriatric syndromes (such as falls, dementia and depression). This situation has prompted the development of research programs aimed at identifying the specific issues faced by this population, including the risk of malnutrition, with a view to identifying appropriate therapeutic responses. Indeed, interventions targeting malnourished older adults yield benefits not only in terms of nutritional status but also reductions in the risk of hospitalisation and complications [21,22]. To the best of our knowledge, no study to date has identified the prevalence of malnutrition and its determinants in older adults living in LTCFs in the French West Indies. Therefore, this study aimed to assess the prevalence of malnutrition and to identify malnutrition-associated variables in older adults living in French Caribbean nursing homes.

## 2. Methods

This is a cross-sectional study of the KASEHPAD (KASEHPAD) study describing the baseline clinical characteristics of older adults living in nursing homes. The KASEHPAD study is a prospective, longitudinal cohort study conducted in the nursing homes for older adults (≥60 years) in Guadeloupe and Martinique. Participants were recruited from 6 of the 41 nursing homes in Guadeloupe and Martinique. To be eligible, residents had to be over 60, live in a nursing home in Guadeloupe or Martinique and be affiliated to the French social security system. The main exclusion criterion was the refusal of the resident or their legal guardian to participate in the study. Participants and their professional caregivers were interviewed by healthcare professionals (geriatric doctors or clinical research nurses) at baseline. The interview comprised a physical evaluation and a cognitive and psychological assessment of the participants. Professional caregivers of the participants provided information about the participant (sociodemographic, medical history, medication, nutrition, level of activity, dependence). The KASEHPAD study is registered under the following RGB-ID: 2020-A00960-39. Version 1.1 (11 May 2020) was approved by the EST 1 French Ethics Committee on 2 June 2020. It was registered at clinicaltrials.gov on 13 October 2020 (NCT04587466). Two amendments were made to the protocol: one to extend the duration of inclusion of residents due to the COVID-19 crisis, and one to include new centres in Martinique (inclusion was initially planned only in Guadeloupe).

### 2.1. Study Design

Setting: The KASEHPAD study is a prospective, observational study of older adults living in nursing homes.

Participants: Participants were recruited among six (6) nursing homes in Martinique and Guadeloupe (French West Indies). Instruments: The interview comprised a physical evaluation and a cognitive and psychological assessment of the participants. The aim of the overall KASEHPAD project is to describe the health trajectories of older adults living in nursing homes for one year. At baseline, 6 months and 12 months, healthcare professionals (geriatricians or clinical research nurses) interviewed the participants and their professional caregivers. For the purpose of the present study, we conducted a cross-sectional analysis of the baseline characteristics of participants. The KASEHPAD study received approval from the French Ethics Committee Est 1 on 2 June 2020 under the number RGB-ID: 2020-A00960-39, and the trial was registered at clinicaltrials.gov on 13 October 2020 under the identifier NCT04587466.

### 2.2. Outcome Measure

The Mini Nutritional Assessment Short-Form (MNA-SF) [23] was used to assess nutritional status. The MNA-SF consists of six items, namely reduced food intake, unintentional weight loss in the past 3 months, a lack of mobility, psychological stress or acute disease during the past 3 months, neuropsychological problems and a low body mass index (BMI). For adults with a missing BMI, it was substituted with a low calf circumference, as recommended in the MNA-SF guidance [23]. The total MNA-SF score ranges from 0 (indicating the most severe form of malnutrition) to 14 (indicating no sign of malnutrition). Specifically, a score of 0–7 indicates malnutrition.

### 2.3. Other Measurements

Demographic data, including age, sex, marital status and total time living in the nursing home, as well as data on morbidity, nutritional status, anthropometry, cognitive function, symptoms of depression and functional status, were collected. The thirty-item Mini-Mental State Examination (MMSE) [24] was used to assess overall cognitive function. The presence of depressive symptoms was assessed using the Center for Epidemiological Studies-Depression (CES-D) scale [25], functional status was assessed using the Activities of Daily Living (ADL) [26], physical function was evaluated using the Short Physical Performance Battery (SPPB) [27] and quality of life was assessed using the EQ-5D-3L. The French threshold value set for the EQ-5D-3L was used to calculate the EQ-5D index value [28,29], with values below zero indicating a health state worse than death and 1 indicating full health.

### 2.4. Statistical Analysis

Data are presented as means ± standard deviations for continuous variables and as numbers and percentages for categorical variables. Chi-square tests or Fisher’s exact tests and Student *t*-tests were used to compare groups according to nutritional status, as appropriate. Logistic regression models were built to examine the association between nutritional status (the independent variable) and comorbidity or geriatric score. Variables that were associated with nutritional status according to the bivariate analysis with a *p*-value < 0.2 were entered into the multivariable logistic regression model. The results are reported as odds ratios (ORs) and 95% confidence intervals (95% CIs). All analyses were performed with R. 4.2.1.

## 3. Results

A total of 332 older adults from six nursing homes were included in the KASEHPAD study. The mean age was 81.3 ± 10.6 years, with half of the participants being men. Among the participants, 319 had an available MNA-SF score. The prevalence of malnutrition (MNA-SF ≤ 7) was 27.6% (95% CI: 22.0–32.5) (*n* = 88). This prevalence increased with age, ranging from 18.0% in individuals aged <75 years to 31.3% in those aged 75 years and older. In the bivariate analysis (Table 1), malnutrition was associated with the following: an older age (84.4 versus 80.3 years; *p* = 0.001), a lower MMSE score (4.8 versus 13.4; *p* < 0.001), a lower ADL score (1.1 versus 2.9; *p* < 0.001), a lower SPPB score (1.0 versus 2.7; *p* < 0.001), a lower EQ5D index score (0.00 versus 0.35; *p* < 0.001), hemiplegia (30.0% versus 6.5%; *p* = 0.003) and a lower number of drugs (5.3 versus 6.2; *p* = 0.022). Malnutrition was not associated with gender, hypertension, diabetes, cardiovascular disease, Parkinson’s disease, kidney disease, chronic pain or cancer.

Table 2 presents the results of the multivariable analysis. A low MMSE score was associated with malnutrition (OR: 0.81 (0.68–0.92); *p* = 0.015) and there was a borderline significant association with a higher CESD score (OR: 1.05 (1.00–1.12); *p* = 0.07).

## 4. Discussion

In this study, the prevalence of malnutrition was estimated at 27.6% among nursing home residents in the French West Indies. This rate is higher than the means reported in other studies among residents of LTCFs. Indeed, Kaiser et al. [28] estimated a prevalence of malnutrition at 13.8%, also using the MNA-SF, which is only half the rate observed in our study. In a review of the literature published in 2016, Bell et al. [29] reported an overall prevalence of malnutrition in nursing homes of 20%, which is also lower than that observed in our study. However, the authors of that review observed wide variability, with reported prevalence ranging from 2 to 60%. This discrepancy compared to our findings could be explained by the profile of the residents (aged 85 years on average in the review, versus 81 years in our study) and the heterogeneity of the articles included in Bell’s review. Moreover, in this review, several different instruments and tests were used to assess nutritional status, including the MNA (full version), MNA-SF, albumin levels, BMI or the Malnutrition Universal Screening Tool (MUST). This could at least partially explain the wide variability in the prevalence rates reported. In another review of a study in the setting of long-term care published in 2023, the prevalence of malnutrition ranged from 6.8 to 75.6%, and the risk of malnutrition was reported to be between 36.5 and 90.4%. The pooled prevalence in individuals with dementia was 26.7% [30]. This finding is more congruent with our observations, which is reasonable given that almost 75% of our population had a major decline in cognitive function (MMSE < 18/30). Another study in nursing homes in Germany also found a prevalence of 26% [31]. 

We found that cognitive decline was associated with malnutrition. This is consistent with the literature. Indeed, as in our study, Jesus P et al. have described a relationship between cognitive disorders and malnutrition in nursing home residents [32]. Weight loss affects 30 to 40% of older persons with moderate to severe Alzheimer’s disease [33,34,35]. The weight loss is often involuntary and appears before the diagnosis of dementia, and may be compounded with disease progression [36,37]. Several mechanisms have been proposed to explain malnutrition in patients with dementia, especially Alzheimer-type dementia. First, a decline in cognitive function can lead to difficulties in providing, preparing and eating meals, as well as appetite disorders [38]. Among subjects with appetite disorders, cerebral imaging often shows medial temporal cortex atrophy [34]. In addition, the dysregulation of appetite can lead to a marked reduction in neuropeptide Y, which is a strong stimulant of food intake and hydration [39]. Alterations in sense of smell, which are often present in older adults with cognitive disorders, will also contribute to appetite disorders. Finally, certain studies have shown that the presence of behavioural disorders, as assessed by the Neuropsychiatric Inventory (NPI) (agitation, apathy, hallucinations, stress, sleep disorders), was more frequent in older subjects with malnutrition [40,41,42].

We observed a borderline significant link between depressive symptoms and malnutrition (*p* = 0.07). A study among the general population of the French West Indies [43] previously reported a link between depressive symptoms and the risk of malnutrition. This relation is bidirectional, as depressive symptoms may be both the cause and the consequence of malnutrition. Indeed, our study precludes any conclusions concerning the causality of this relation, and the lack of statistical significance could be due to a lack of statistical power. Indeed, in our population of nursing home residents, almost 75% had severe cognitive decline, as defined by an MMSE score < 18, and only 121 participants had an available CES-D score. Weight loss, which is one of the criteria assessed in the MNA, is directly cited as a possible depressive symptom in the diagnostic criteria of the Diagnostic and Statistical Manual of Mental Disorders, Fifth Edition (DSM-5). This is explained by the direct link with depressive symptoms, which include loss of appetite, asthenia and a loss of motivation or pleasure in daily activities. Some diseases can cause both malnutrition and depressive symptoms, and depression itself can induce neuroendocrine changes that disturb the regulation of appetite and food intake [6].

In our study, we did not observe an association between pain and malnutrition, although such a link has previously been reported in studies in the setting of LTCFs [44]. A possible explanation for this is the improved management of pain in nursing homes in recent years. Healthcare professionals working in LTCFs are more aware of the problem of pain, with improved training and the availability of “pain protocols” in many LTCFs. From a clinical point of view, to optimize the management of malnutrition in nursing home residents, targeted training is warranted, focusing on the specificities of older persons. This may include managing a loss of appetite, communication difficulties, mood disorders, swallowing disorders and neurocognitive or behavioural disorders that can be observed at mealtimes. A recent study reported that not having a guideline on the prevention and treatment of malnutrition and the absence of a dietician in the nursing home were factors associated with a higher prevalence of weight loss [45]. It appears crucial to implement protocols to monitor nutrition in nursing home residents, especially those with severe cognitive disorders. 

To the best of our knowledge, this is the first study to investigate malnutrition in the setting of LTCFs in the Caribbean region. The quality of the data recording process was ensured by dedicated research nurses specifically trained in the implementation of geriatric evaluation tools. Our population accounts for 10% of the total population of nursing home residents in the French West Indies. Our study is also limited by its design: the observational design precludes any identification of causality. 

Our results provide valuable insights for future strategies to manage malnutrition in older individuals. Preserving an adequate nutritional status during aging, with or without illness, is fundamental and of major prognostic importance. 

## 5. Conclusions

In this study among nursing homes in the French West Indies, we found that cognitive decline was significantly associated with an increased risk of malnutrition. Symptoms of depression also appear to be borderline associated with malnutrition. Although we cannot establish the causality of this relationship, the detection of these three geriatric syndromes among older adults living in LTCFs is crucial for the prevention of adverse health outcomes. Further studies are warranted to confirm and consolidate our findings.

## Figures and Tables

**Table 1:** Comparison of population characteristics according to nutritional status.

	KASEHPAD Cohort (*n* = 332)	Participants with Available MNA-SF Score (*n* = 319)		
Characteristics	All	Malnutrition Yes (*n* = 88)	Malnutrition No (*n* = 231)	*p*
Age	81.3 ± 10.6	84.4 ± 9.3	80.3 ± 10.1	0.001
Age < 75 years	237 (71.4%)	16 (18.0%)	73 (31.6%)	0.017
Age ≥ 75 years	95 (28.6%)	72 (81.8%)	158 (68.4%)	
Men	168 (50.6%)	39 (44.3%)	121 (52.4%)	0.198
Hypertension	221 (66.6%)	55 (62.5%)	159 (68.8%)	0.282
Diabetes	94 (28.3%)	19 (21.6%)	71 (30.7%)	0.105
Hypercholesterolemia (*n* = 330)	53 (16.1%)	4 (13.3%)	7 (9.1%)	0.498
Myocardial infarction (*n* = 330)	11 (3.3%)	1 (1.2%)	9 (3.9%)	0.291
Cardiac failure (*n* = 330)	34 (10.2%)	8 (9.3%)	25 (10.8%)	0.693
Stroke	54 (16.3%)	15 (17.0%)	38 (16.4%)	0.898
Dementia	173 (52.1%)	65 (73.9%)	105 (45.4%)	<0.001
Parkinson’s disease	30 (12.2%)	8 (9.1%)	18 (7.8%)	0.705
Kidney disease	48 (14.8%)	8 (9.6%)	38 (16.7%)	0.122
Cancer	14 (4.2%)	4 (4.5%)	9 (3.9%)	0.758
Chronic pain	143 (43.2%)	43 (49.4%)	95 (41.1%)	0.183
Number of drugs	5.9 ± 3.0	5.3 ± 2.8	6.2 ± 3.1	0.022
ADL score (*n* = 326)	2.4 ± 2.1	1.1 ± 1.6	2.9 ± 2.1	<0.001
MMSE score (*n* = 295)	11.3 ± 9.4	4.8 ± 1.6	13.4 ± 9.3	<0.001
SPPB score (*n* = 309)	2.3 ± 3.3	1.0 ± 2.0	2.7 ± 3.5	<0.001
CESD score (*n* = 144)	15.7 ± 14.4	22.3 ± 15.8	14.8 ± 14.2	0.05
EQ5D index (*n* = 314)	0.25 ± 0.41	0.00 ± 0.34	0.35 ± 0.4	<0.001

ADL: Activities of Daily Living, MMSE: Mini-Mental State Examination, SPPB: Short Physical Performance Battery, CESD: Center for Epidemiological Studies-Depression.

**Table 2:** Factors associated with risk of malnutrition according to multivariable logistic regression.

Variable	Estimate	*p*	OR (95% CI)
Age	0.01	0.144	-
Gender (men)	−1.36	0.144	-
Diabetes	−0.18	0.993	-
Kidney disease	−0.17	0.995	-
Chronic pain	0.87	0.345	-
Number of drugs	0.01	0.953	-
MMSE score	−0.29	0.015	0.81 (0.68–0.92)
ADL score	0.14	0.749	-
SPPB score	0.01	0.962	-
CESD score (*n* = 121)	0.052	0.070	1.05 (1.00–1.12)
EQ5D index	1.44	0.538	-

ADL: Activities of Daily Living, MMSE: Mini-Mental State Examination, SPPB: Short Physical Performance Battery, CESD: Center for Epidemiological Studies-Depression. McFadden’s Pseudo R2: 0.882.

## Data Availability

The datasets used and/or analysed during the current study are available from the corresponding author on reasonable request.
